# Primary care dental professionals' experiences of sharp injuries in Qatar: A cross-sectional study

**DOI:** 10.3389/froh.2022.1014004

**Published:** 2022-12-02

**Authors:** Tamara Marji, Mohamed Ahmed Syed

**Affiliations:** Department of Clinical Research, Directorate of Clinical Affairs, Primary Health Care Corporation, Doha, Qatar

**Keywords:** sharp injuries, primary care, general practice, dentists, dental assistants, Qatar, needle stick injuries, occupational health

## Abstract

**Objectives:**

Very few studies assess sharp injuries solely among dental professionals globally. This study aims to understand the experiences of sharp injuries among primary care dental professionals in Qatar.

**Methods:**

This is a cross-sectional study where dental professionals working at the Primary Health Care Corporation (PHCC), a public sector healthcare organization and Qatar's largest primary care provider with 27 primary healthcare centers in Qatar, were invited to participate. An online questionnaire was administered to understand participants' experience of sharp injuries and access to occupational health services and training.

**Findings:**

A total of 114 dental professionals participated in this study. In total, 68.42% (*N*=78) of participants reported experiencing a sharp injury in the 12 months prior to the survey. Majority (*N*=58; 75.36%) of the reported causes of sharp injuries were related to dental explorers (*N*=31; 40.26%) and dental injection needles (*N*=27; 40.26%). Of those who had experienced a sharp injury, 84.47% (*N*=87) did not report them.

**Conclusion:**

In conclusion, the results highlight that sharp injuries are common among primary care dental professionals, and despite having good access to occupational support services, most dental professionals did not report their injuries. Continuous education and training programs must be implemented to improve knowledge and raise awareness to reduce the burden of sharp injuries among dental professionals. More studies from other dental settings are needed to better understand dental professionals' experiences of sharp injuries in Qatar and elsewhere.

## Introduction

Due to the nature of their work, healthcare workers (HCWs) in proximity to infected patients are susceptible to direct (e.g., through airborne transmission) or indirect (contamination of instruments or surfaces) transmission of infection (1). Sharps, defined as any instrument that has the potential to cause a penetrating injury to the skin, are the most common sources of infection among HCWs (2). Blood-borne pathogens, such as the human immunodeficiency virus (HIV), hepatitis B virus (HBV), hepatitis C virus (HCV), and *Treponema pallidum*, can be transmitted from these injuries (3–5). The risk of transmission after sustaining a pathogen-positive sharp injury is estimated to be 0.3% for HIV (6), 6.0%–30.0% for HBV (7), and 0%–10.0% for HCV (4, 8–10).

Approximately 3 out of 35 million HCWs worldwide experience sharp injuries annually, exposing them to blood-borne pathogens (2, 11). A meta-analysis reported 1-year global pooled sharp injuries of 44.5% among HCWs. Sharp injuries pose a major occupational hazard for dental professionals due to the routine use of sharps and exposure to blood and saliva in the oral cavity. Sharp injuries have been reported to be the highest among dental professionals, compared with other healthcare professionals, at 59.1% (12). Therefore, sharp injuries pose a serious public health problem in contemporary dentistry.

Very few studies assess sharp injuries solely among dental professionals globally (13). Previous studies have shown that most sharp injuries are preventable, and information related to the circumstances in which they occur is helpful in finding preventive measures (14). To date, no studies report on this important public health issue in Qatar, which has 1,741 dentists and 799 dental assistants (Global Health Workforce Statistics, 2018). This study aims to address this gap in knowledge. The findings of the study will help to understand the problem and implement strategies to prevent sharp injuries in Qatar's dental primary care settings.

## Methodology

This is a cross-sectional study. Dental assistants and dentists working at Primary Health Care Corporation (PHCC), a public sector healthcare organization and Qatar's largest primary care provider with 27 primary healthcare centres in Qatar were invited to participate in the study.

The study questionnaire was adapted from a questionnaire used in a previously published study from the United Kingdom (13). It consisted of participant demographic questions (age, gender, and clinical role), participants' experience of sharp injuries in the past 12 months (how many, most common reason for injury, and the type of anaesthesia syringe used in practice), whether they reported sharp injuries in the past 12 months or not, and the reasons for not reporting. Participants were also asked if they sought medical advice for their sharp injury and whether they had access to occupational health services and training in the past 12 months. Participants were invited to participate in the study by sending an email to their work email address, which included information about the study and an online link to complete the questionnaire. Invitation emails were sent in May 2021. Two reminders were sent (one per week). Participants were informed that participation is voluntary. All responses were recorded anonymously.

Questionnaire responses were analysed using the “Statistical Package for the Social Sciences” statistical software package. Basic descriptive statistics were used to analyse participant demographics (age, gender, and clinical role), experience of sharp injuries, and access to occupational health services and training data. Chi-squared/Fisher's exact tests were used to establish associations with a *p*-value of below 0.05 for significance.

## Results

A total number of 190 dental assistants and 216 dentists working at PHCC were invited to participate in this study. 31.6% (*N*=60) dental assistants and 25% (*N*=54) dentists completed the questionnaire. The overall response rate was 28%.

General demographic details of the participants are presented in Table 1. About 52.6% (*N*=60) of the participants were dental assistants, and the rest were dentists. The majority of the participants were 35–44 years old (*N*=48; 42.1%). About 72.8% (*N*=83) of the participants were women.

**Table 1:** Participant demographic characteristics.

	*N*	Percentage
Age	25–34	21	18.4
	35–44	48	42.1
	45–54	33	28.9
	55–64	11	9.6
	>65	1	0.9
	Total	114	100
Gender	Woman	83	72.8
	Man	31	27.2
	Total	114	100
Clinical role	Dental assistant	60	52.6
	Dentist	54	47.4
	Total	114	100

A total of 90 participants reported experiencing a sharp injury in the 12 months prior to the survey (Table 2). Both dentists and dental assistants experienced sharp injuries. About 57.02% (*N*=65) participants have reported one injury, and 11.4% (*N*=13) reported two or more injuries.

**Table 2:** Experience of sharps injuries in the past 12 months.

	Dental assistant		Dentist		Totals		*p*-Value
	*N*	Percentage	*N*	Percentage	*N*	Percentage	
Number of sharp injuries							
0	16	14.04	20	17.54	36	31.58	0.194
1	37	32.46	28	24.56	65	57.02	
2	1	0.88	5	4.39	6	5.26	
3	3	2.63	0	0	3	2.63	
5	1	0.88	0	0	1	0.88	
>5	2	1.75	1	0.88	3	2.63	
Total	60	52.63	54	47.37	114	100	
Common cause of injury							
Dental injection needle	9	11.69	18	23.38	27	35.06	0.003
Dental explorer	24	31.17	7	9.09	31	40.26	
Reamer/file	5	6.49	4	5.19	9	11.69	
Disposable irrigation needle	4	5.19	1	1.30	5	6.49	
Scissors	1	1.30	0	0.00	1	1.30	
Extraction forceps	0	0	1	1.30	1	1.30	
Cutting instruments	0	0	3	3.90	3	3.90	
Total	43	55.84	34	44.16	77	100	
Type of dental anaesthesia syringes used							
Non-safety device	10	8.93	12	10.71	22	19.64	0.366
Device with sharps safety	50	44.64	40	35.71	90	80.36	
Total	60	53.57	52	46.43	112	100	
Reporting of sharps injury							
Yes	10	9.71	6	5.83	16	15.53	0.451
No	45	43.69	42	40.78	87	84.47	
Total	55	53.40	48	46.60	103	100	
Reason for not reporting							
Did not consider patient to be high risk	1	1.35	1	1.35	2	2.70	0.754
Sterile or clean needle stick	22	29.73	22	29.73	44	59.46	
Low perception of risk	4	5.41	3	4.05	7	9.46	
Lack of time	1	1.35		0.00	1	1.35	
Concerns about confidentiality and professional discrimination	1	1.35	1	1.35	2	2.70	
Excessive paperwork	0	0	1	1.35	1	1.35	
Not familiar with reporting process	0	0	2	2.70	2	2.70	
Other	9	12.16	6	8.11	15	20.27	
Total	38	51.35	36	48.65	74	100	

The majority (*N*=58; 75.36%) of the reported causes of sharp injuries were related to dental explorers (*N*=31; 40.26%); and dental injection needles (*N*=27; 40.26%). Among dental assistants, dental explorer (*N*=24; 31.17%) was the most common cause, while dental injection needle was the most common cause (*N*=18; 23.38%) for dentists. There are 80.36% of participants who reported using a device with sharp safety.

Of those who had experienced a sharp injury, 84.47% (*N*=87) did not report it. Sterile or clean needle was the most commonly reported reason (*N*=44; 59.46%), followed by the low perception of risk (*N*=7; 9.46%).

About 31.63% (*N*=31) of the participants sought medical advice to immediately manage their injury (Table 3). The majority (*N*=30; 96.77%) of those who experienced an injury sought advice from PHCC. Further, 75.93% (*N*=82) reported they had access to occupational health support, and 37.6% (*N*=42) stated they had received sharp injury prevention training in the 12 months prior to the survey.

**Table 3:** Medical advice and access to occupational health service and training.

	Dental assistant		Dentist		Totals		*p*-Value
	*N*	Percentage	*N*	Percentage	*N*	Percentage	
Sought medical advice to immediately manage injury							
Yes	15	15.31	16	16.33	31	31.63	0.577
No	37	37.76	30	30.61	67	68.37	
Total	52	53.06	46	46.94	98	100	
Where medical advice was sought from							
Hamad Medical Corporation	1	3.23	0	0	1	3.23	0.741
Primary Health Care Corporation	14	45.16	16	51.61	30	96.77	
Other	0	0	0	0	0	0	
Total	15	48.39	16	51.61	31	100	
Access to occupational health support in the last 12 months							
Yes	49	45.37	33	30.56	82	75.93	0.002
No	0	0	7	6.48	7	6.48	
Total	58	53.70	50	46.30	108	100	
Had training on the prevention of sharps injury in the last 12 months							
Yes	20	18.18	22	20.00	42	38.18	0.384
No	39	35.45	29	26.36	68	61.82	
Total	59	53.64	51	46.36	110	100	

## Discussion

Many studies have reported virus infection and transmission, the prevalence and causes of occupational injuries, and the prevention among healthcare workers in general (3, 4, 12, 14, 15); however, few have addressed the prevalence and experience of sharp injuries in dental professionals (1, 5, 13). In Qatar, there are no studies related to sharp injuries among dental professionals. This is the first study to report such findings in a primary care setting. The study found sharp injuries are common (*N*=78; 68.42%), and a significantly high proportion of dental professionals did not report their injuries (*N*=87; 84.47%) despite having access to occupational health support.

In general, dental professionals experience a high prevalence of sharp injuries due to their inevitable work conditions; they work with sharp instruments in a relatively limited workspace (15–21). In this study, the most commonly reported causes of sharp injuries were related to dental explorers and dental injection needles. There are 80.36% (*N*=90) of the study participants that said they used dental safety syringes. However, it appears that they are not effective in preventing sharp injuries caused by dental syringes. It may be related to the inadequate safety engineering of the devices used or training.

In this study, dental professionals have reported having access to occupational health support (*N*=82; 75.93%). Nevertheless, only 84.47% (*N*=87) did not report their injuries. The most common reason (*N*=44; 59.46%) for the not reported injuries was stated to be injuries related to a sterile or clean needle stick. These findings are in line with those reported by Savić Pavičin et al. (20) and Bellissimo-Rodrigues et al. (21) wherein 83.5% and 90.5% of dental professionals, respectively, did not seek the help of a doctor because of their injuries. In another study by Aldakhil et al. (1), only 19% of the dental assistants received post-exposure prophylaxis for their injury. An explanation as to why dental professionals do not routinely report sharp injuries could be explained by their low perception of the risk of such injuries, a lack of knowledge, or even difficulties in reporting them. Acquiring injuries with a sterile needle or sharp object may not affect the dental worker themselves, but it still poses a danger to patients if it is still used (20–23); therefore, continuous education about the risk of acquiring infections from such injuries is highly recommended (22).

Key strengths of the study are the use of a validated tool that was administered to primary care dental professionals. However, its limitations include a low response rate. It could be attributed to the time constraints and high workload, besides believing that research studies will not help improve working conditions (23). Another limitation is the recall bias of events that occurred sometime prior to the study (22).

## Conclusions

In conclusion, the results highlight that sharp injuries are common among primary care dental professionals. Dental injection needles account for a large proportion of sharp injuries, despite the wide use of devices with sharp safety. This indicates the lack of effectiveness of devices in preventing sharp injuries. They must be replaced by better-engineered alternatives if available.

Furthermore, despite having good access to occupational support services, the majority of dental professionals did not report their injuries. Continuous education and training programmes must be implemented to improve and raise awareness to reduce the burden of sharp injuries among dental professionals.

The results of this study can help establish clear and uniform policies for primary care dental professionals to create safer work environments across Qatar. More studies from other dental settings are needed to improve understanding of dental professionals' experiences of sharps injuries in Qatar and elsewhere.

## Data Availability

The data that support the findings of this study are available upon reasonable request from the corresponding author. The data are not publicly available due to privacy or ethical restrictions.

## References

[1] AlDakhil L Yenugadhati N Al-Seraihi O Al-Zoughool M. Prevalence and associated factors for needlestick and sharp injuries (NSIs) among dental assistants in Jeddah, Saudi Arabia. Environ Health Prev Med. (2019) 24(1):60. 10.1186/s12199-019-0815-7 31601166 PMC6788026

[2] World Health Organization. *The World health report 2002: reducing risks, promoting healthy life: overview*. WHO/WHR/02.1 (2002).

[3] Gerberding JL. Incidence and prevalence of human immunodeficiency virus, hepatitis B virus, hepatitis C virus, and cytomegalovirus among health care personnel at risk for blood exposure: final report from a longitudinal study. J Infect Dis. (1994) 170(6):1410–7. 10.1093/infdis/170.6.1410 7995979

[4] Lanphear BP Linnemann CC Jr. Cannon CG DeRonde MM Pendy L Kerley LM. Hepatitis C virus infection in healthcare workers: risk of exposure and infection. Infect Control Hosp Epidemiol. (1994) 15(12):745–50. 10.2307/30148418 7534324

[5] Shah SM Merchant AT Dosman JA. Percutaneous injuries among dental professionals in Washington State. BMC Public Health. (2006) 6:269. 10.1186/1471-2458-6-269 17074095 PMC1635049

[6] Henderson DK Fahey BJ Willy M Schmitt JM Carey K Koziol DE Risk for occupational transmission of human immunodeficiency virus type 1 (HIV-1) associated with clinical exposures. A prospective evaluation. Ann Intern Med. (1990) 113(10):740–6. 10.7326/0003-4819-113-10-740 2240876

[7] Mauskopf JA Bradley CJ French MT. Benefit–cost analysis of hepatitis B vaccine programs for occupationally exposed workers. J Occup Med. (1991) 33(6):691–8. 10.1097/00043764-199106000-00009 1824040

[8] Mitsui T Iwano K Masuko K Yamazaki C Okamoto H Tsuda F Hepatitis C virus infection in medical personnel after needlestick accident. Hepatology. (1992) 16(5):1109–14. 10.1002/hep.1840160502 1427651

[9] Kiyosawa K Sodeyama T Tanaka E Nakano Y Furuta S Nishioka K Hepatitis C in hospital employees with needlestick injuries. Ann Intern Med. (1991) 115(5):367–9. 10.7326/0003-4819-115-5-367 1907441

[10] Hernandez ME Bruguera M Puyuelo T Barrera JM Sanchez Tapias JM Rodés J. Risk of needle-stick injuries in the transmission of hepatitis C virus in hospital personnel. J Hepatol. (1992) 16(1-2):56–8. 10.1016/S0168-8278(05)80094-7 1484168

[11] US Department of Health and Human Services PHS, Centers for Disease Control and Prevention, National Institute for Occupational Safety and Health. *Preventing needlestick injuries in health care settings*. In: Health NIfOSa, editor. DHHS (NIOSH) Publication Number. Cincinnati, OH: National Institute for Occupational Safety and Health. p. 28).

[12] Bouya S Balouchi A Rafiemanesh H Amirshahi M Dastres M Moghadam MP Global prevalence and device related causes of needle stick injuries among health care workers: a systematic review and meta-analysis. Ann Glob Health. (2020) 86(1):35. 10.5334/aogh.2698 32346521 PMC7181946

[13] Trayner KMA Hopps L Nguyen M Christie M Bagg J Roy K. Cross-sectional survey of a sample of UK primary care dental professionals' experiences of sharps injuries and perception of access to occupational health support. Br Dent J. (2018) 225:1023–8. 10.1038/sj.bdj.2018.1031 30499564

[14] Cullen BL Genasi F Symington I Bagg J McCreaddie M Taylor A Potential for reported needlestick injury prevention among healthcare workers through safety device usage and improvement of guideline adherence: expert panel assessment. J Hosp Infect. (2006) 63(4):445–51. 10.1016/j.jhin.2006.04.008 16777264

[15] Malik A Shaukat MS Qureshi A. Needle-stick injury: a rising bio-hazard. J Ayub Med Coll Abbottabad. (2012) 24(3-4):144–6. 24669637

[16] Ramaswami E Nimma V Jakhete A Lingam AS Contractor I Kadam S. Assessment of occupational hazards among dentists practicing in mumbai. J Family Med Prim Care. (2020) 9(4):2016–21. 10.4103/jfmpc.jfmpc_1180_19 32670958 PMC7346943

[17] Pervaiz M Gilbert R Ali N. The prevalence and underreporting of needlestick injuries among dental healthcare workers in Pakistan: a systematic review. Int J Dent. (2018) 2018:9609038. 10.1155/2018/9609038 29623091 PMC5829343

[18] Kapoor V Gambhir RS Singh S Gill S Singh A. Knowledge, awareness and practice regarding needle stick injuries in dental profession in India: a systematic review. Niger Med J. (2013) 54(6):365–70. 10.4103/0300-1652.126283 24665148 PMC3948956

[19] Younai FS Murphy DC Kotelchuck D. Occupational exposures to blood in a dental teaching environment: results of a ten-year surveillance study. J Dent Educ. (2001) 65(5):436–48. 10.1002/j.0022-0337.2001.65.5.tb03413.x 11425248

[20] Savić Pavičin I Lovrić Ž Zymber Çeshko A Vodanović M. Occupational injuries among dentists in Croatia. Acta Stomatol Croat. (2020) 54(1):51–9. 10.15644/asc54/1/6 32523157 PMC7233126

[21] Bellissimo-Rodrigues WT Bellissimo-Rodrigues F Machado AA. Occupational exposure to biological fluids among a cohort of Brazilian dentists. Int Dent J. (2006) 56(6):332–7. 10.1111/j.1875-595X.2006.tb00337.x 17243465

[22] Khader Y Burgan S Amarin Z. Self-reported needle-stick injuries among dentists in north Jordan. East Mediterr Health J. (2009) 15(1):185–9. 10.26719/2009.15.1.185 19469442

[23] Leavy P Templeton A Young L McDonnell C. Reporting of occupational exposures to blood and body fluids in the primary dental care setting in Scotland: an evaluation of current practice and attitudes. Br Dent J. (2014) 217(4):E7. 10.1038/sj.bdj.2014.734 25146830

